# The Touro 12-Step:  A Systematic Guide to Optimizing Survey Research with Online Discussion Boards

**DOI:** 10.2196/jmir.1314

**Published:** 2010-05-27

**Authors:** Eric J Ip, Mitchell J Barnett, Michael J Tenerowicz, Paul J Perry

**Affiliations:** ^1^Touro University College of PharmacyVallejoUnited States

**Keywords:** Anabolic Steroids, Internet Research

## Abstract

The Internet, in particular discussion boards, can provide a unique opportunity for recruiting participants in online research surveys.  Despite its outreach potential, there are significant barriers which can limit its success.  Trust, participation, and visibility issues can all hinder the recruitment process; the Touro 12-Step was developed to address these potential hurdles.  By following this step-by-step approach, researchers will be able to minimize these pitfalls and maximize their recruitment potential via online discussion boards.

## A Proposed Process for Posting Surveys in Discussion Boards

The Internet, particularly online discussion boards, can be a useful and low cost instrument in recruiting participants for online surveys and data collection [1,2]. Online discussion boards often provide quick access to hundreds or even thousands of participants with similar interests within a relatively short period of time [1]. Furthermore, discussion board users generally encompass diverse geographical (often worldwide) and demographic segments of the study population universe and can be useful in facilitating and streamlining the recruitment process [3,4]. Multiple studies and a review by Krantz and Dalal have shown that web-based data collection and traditional methods (e.g. paper and pencil) result in equivalent conclusions, demonstrating the validity and reliability of online data collection for research [5,6,7,8].

Despite the tremendous potential, recruitment of subjects via online discussion boards may not be an easy task [9,10]. A study by Koo and Skinner describes the struggles in obtaining subjects from online discussion boards including: 1) Survey postings being immediately removed by a board administrator; 2) Messages and survey links being mistaken for “spam,” and 3) Having poor visibility on the discussion forums within a few days of the initial post [9]. As a result, the authors expressed disappointment in the small number of subjects recruited for a study. Presented here are insights and strategies to address these issues. These insights were gained from experience developing a systematic approach to successfully recruit study participants via online discussion boards.

A clinical research team at Touro University-CA College of Pharmacy in Vallejo, CA utilized various bodybuilding, weightlifting, fitness, and anabolic steroid discussion forums to recruit subjects to participate in an online research survey (Touro University-CA IRB# P-0308). Since many of the discussion boards approached require registration to gain access, an informed consent page was created to deal with potential privacy issues, to assure confidentiality and anonymity, and to provide additional information regarding the study [11]. Clearly stated on the informed consent page were the disclosures that no individually identifiable data would be collected, that internet provider (IP) addresses would not be logged, and that all data transfer would be encrypted. After consenting to take part in the study, the strength-trained subject was allowed to start the 99-item survey which queried specific information related to exercise trends, medication utilization, behavior/psychiatric traits, and demographic variables.

Between February and June 2009, the survey was posted on over 50 different discussion boards with varying success. Encountered initially were several limitations resulting in low survey attempt and completion rates. These limitations included: 1) Postings and survey link being quickly removed by website administrators; 2) Lack of initial trust from discussion board members; 3) Lack of enthusiasm by members for participation in a survey with no apparent reward; and 4) Losing visibility of the thread and survey link when it was no longer on the first page of the discussion board forums. After encountering these hurdles during the early phases of enrollment, a systematic step-by-step (12-step) process was developed to improve the popularity and visibility of the survey link on respective sites. This 12-step approach led to a marked increase in survey attempts and completion rates. Using this 12-step method, the link was successfully posted on over 30 individual sites, resulting in over 2,250 survey attempts generated worldwide with over 1,500 individuals (518 admitted anabolic steroid users) completing the survey during a relatively short four-month window (February-June, 2009).

### The Touro 12-Step Process

1. Use an internet search engine (e.g. Google.com) to search for websites that have discussion boards which suit your study’s topic (e.g. “bodybuilding forums,” “weightlifting forums,” “steroid discussion boards”).
2. When an appropriate website discussion board is found, determine if the discussion board has an adequate number of members/views/activity.
3. Sign up as a member of that discussion board (create a user name and password).
4. Look for a discussion section that is most appropriate to introduce the survey (e.g. “Bodybuilders,” “Powerlifting,” “Anabolic Steroid Discussion,” “Female Bodybuilders”).
5. Create a simple yet accurate title for the thread (e.g. “Exercise Study” or “Steroid Survey”).
6. Post an introduction thread that explains the research objectives and facilitates feedback/questions from the discussion board users. Include the actual name and credentials of the researcher involved, but avoid using the prefix “Dr.” as this may appear less personable. It should be emphasized: *Do not include the research survey link in the first post.* Website moderators and members often do not trust a researcher who is a first-time poster and may even perceive that individual as an outsider or an “intruder,” potentially altering the discussion board environment [11]. At best, the thread may be removed—and there is a likely chance that your username and IP address will be permanently banned from the website. It is important to develop a rapport with the website members and administrators before attempting to post the survey link.
7. Subscribe to the created thread so that instant e-mail notification can be received anytime a website member posts a response. Timely responses (ideally within 12-24 hours) are valuable as this demonstrates to other website members the seriousness and willingness to address their concerns.
8. Only post the survey link when support of the discussion board members and moderators has been clearly established. This will increase the chances of having a high participation rate and prevent the survey link from being prematurely removed.
9. Create an active and ongoing discussion. Asking board members questions and soliciting feedback will create enthusiasm about the research topic and survey.
10. As days and weeks transpire, answering posts from members provides two benefits: a) continuing to increase interest in the survey and b) “bumping” or moving the survey thread back to the top of the discussion board (improving visibility of the thread).
11. Be courteous. Thank participants when they make a post stating that they have completed the survey (e.g. “Thanks for supporting our survey!”). Website members appreciate the politeness and just as importantly, the “thank you” post will bring the thread back to the top of the discussion board forum (again improving visibility).
12. Don’t go overboard. If there has been no activity or replies on the thread, wait at least 5-10 days before reposting (more frequent attempts to promote the survey may become an annoyance to discussion board members). Some sites may be fine with “bumping” or promoting survey participation more frequently, so pay attention and acquire a feel for the particular forum group. Try to provide value when reposting to move the thread back to the top (e.g. post progress on survey participation or provide an update on reaching the survey recruitment goal). This is especially useful towards the end of data collection to create a strong, final push.

Using the internet, especially online discussion boards, to collect survey data can be very powerful and a cost-efficient tool to promote your research survey. Over 1.5 billion individuals, roughly 23.8% of the world’s population, utilize the internet on a regular basis [12]. To help maximize its recruiting potential, it is imperative to recognize and address potential challenges. Keys to success are to find website forums that suit the research needs, to develop a rapport with website members and moderators, to post the survey link at an appropriate time, and to strategically increase the survey link visibility through reposting and responding to website members. Finally, when the results have been compiled and are finally ready to be reported for publication, it is recommended to follow the CHERRIES (Checklist for Reporting Results of Internet E-Surveys) guidelines to ensure quality and thoroughness [13]. By utilizing the Touro 12-Step, researchers may be able to increase recruiting potential with online discussion boards.
